# Trend analysis and modelling of gender-specific age, period and birth cohort effects on alcohol abstention and consumption level for drinkers in Great Britain using the General Lifestyle Survey 1984–2009

**DOI:** 10.1111/add.12330

**Published:** 2013-09-13

**Authors:** Yang Meng, John Holmes, Daniel Hill-McManus, Alan Brennan, Petra Sylvia Meier

**Affiliations:** School of Health and Related Research, University of SheffieldSheffield, UK

**Keywords:** Abstention, age–period–cohort modelling, alcohol, time–series, trends

## Abstract

**Background and aims:**

British alcohol consumption and abstinence rates have increased substantially in the last 3 decades. This study aims to disentangle age, period and birth cohort effects to improve our understanding of these trends and suggest groups for targeted interventions to reduce resultant harms.

**Design:**

Age, period, cohort analysis of repeated cross-sectional surveys using separate logistic and negative binomial models for each gender.

**Setting:**

Great Britain 1984–2009.

**Participants:**

Annual nationally representative samples of approximately 20 000 adults (16+) within 13 000 households.

**Measurements:**

Age (eight groups: 16–17 to 75+ years), period (six groups: 1980–84 to 2005–09) and birth cohorts (19 groups: 1900–04 to 1990–94). Outcome measures were abstinence and average weekly alcohol consumption. Controls were income, education, ethnicity and country.

**Findings:**

After accounting for period and cohort trends, 18–24-year-olds have the highest consumption levels (incident rate ratio = 1.18–1.15) and lower abstention rates (odds ratio = 0.67–0.87). Consumption generally decreases and abstention rates increase in later life. Until recently, successive birth cohorts' consumption levels were also increasing. However, for those born post-1985, abstention rates are increasing and male consumption is falling relative to preceding cohorts. In contrast, female drinking behaviours have polarized over the study period, with increasing abstention rates accompanying increases in drinkers' consumption levels.

**Conclusions:**

Rising female consumption of alcohol and progression of higher-consuming birth cohorts through the life course are key drivers of increased per capita alcohol consumption in the United Kingdom. Recent declines in alcohol consumption appear to be attributable to reduced consumption and increased abstinence rates among the most recent birth cohorts, especially males, and general increased rates of abstention across the study period.

## Introduction

**Figure 1:**
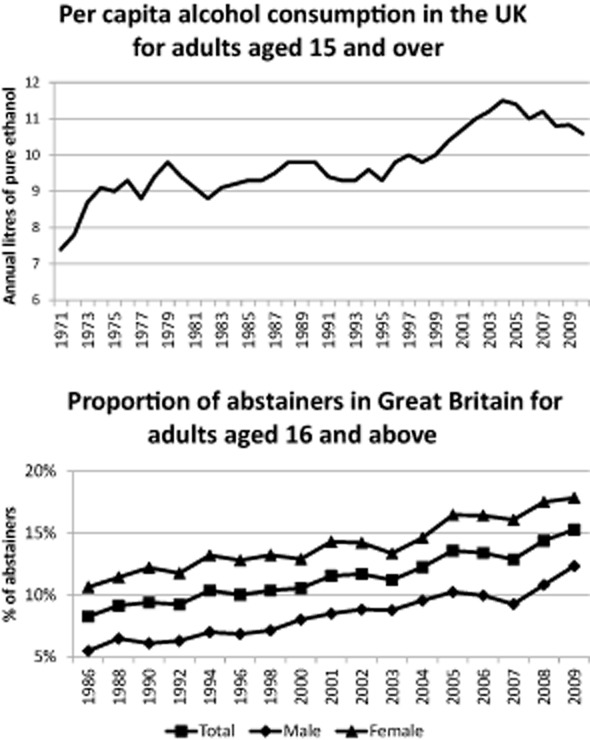
Trends in adult per capita alcohol consumption and proportion of abstainers in the United Kingdom

Rising UK alcohol consumption in the second half of the 20th century 1 has led to substantial policy debate. Sales and duty data show annual per capita alcohol consumption in the United Kingdom for adults aged 15 years and over increased from 7.4 litres of pure alcohol in 1971 to a peak of 11.5 litres in 2004, before falling to 10.8 litres in 2008 2 (Fig. 1). Other sources show this decline has continued to 2010 3. Attempts to explain the origins of these trends have tended to implicate behaviour change affecting specific demographic groups within the population, notably rising youth consumption 4 and, to a lesser extent, increased female consumption (e.g. 5).

Paradoxically, rates of abstinence have also increased markedly (Fig. 1) and, although there has been less research concerning this trend, the focus has again been on young people and the reasons for rising abstinence in this age group 5,6. Evidence for such demographically focused explanations of population trends can often be found within descriptive analyses of survey data (e.g. 7); however, such analyses cannot separate changes straightforwardly within subgroups from population-wide change. For example, it is unclear whether the reported rise in female consumption is driven primarily by changes occurring across society, within youth behaviour or the progression of increasingly high-consuming female birth cohorts through the life-course.

Previous studies of the origins of trends in population-level alcohol consumption can provide some insight into these questions. Lederman proposed that, on average, populations tend to change their behaviour in concert; ensuring regularity can be observed in the relationship between mean consumption and levels of harmful consumption over time 8. Although widely challenged (e.g. 9,10), Lederman's hypothesis is largely supported by Skog's influential theory of collectivity within drinking cultures 11. This proposed a model of behaviour change whereby consumption trends have a ‘central tendency’ 12 to result from individuals and groups shifting their behaviour and then these changes accumulating subsequently into long waves of gradual population-level effects as they spread via social networks and impact across society. Other work has focused on how changing behaviour within specific groups can contribute to the population trend. This includes the GENACIS (Gender, Alcohol and Culture: An International Study) project's analyses of changing female consumption patterns in Europe 13 and focused studies of particular age groups such as the ESPAD (European School Survey Project on Alcohol and Other Drugs) 14.

Although useful, much of this literature does not consider different demographic components of change simultaneously. A small number of age, period and cohort (APC) studies using time–series of cross-sectional consumption survey data address this point. APC studies seek to separate the population trends into three types of demographic trend: (i) trends across the life-course (age effects); (ii) trends across the whole population over time (period effects) and (iii) trends across successive generations (cohort effects). Ideally, this is achieved through statistical modelling to isolate APC effects from each other; however, descriptive analyses are also possible. In the United Kingdom, Kemm descriptively analysed rates of low and heavy consumption within gender-specific birth cohorts from 1978 to 1998 and found that heavy consumption rates declined with age and rose in more recent birth cohorts 15. International APC studies have been carried out in the United States 16–20 and various European countries 21–24, with analyses covering a broad range of outcome measures (e.g. per capita total and beverage-specific consumption 16,17,19–21, rates of problem and binge drinking 16,18,20,22,23, and rates of abstention 15,21,24). Results show that in most contexts consumption peaks in early adulthood, but period and cohort effects vary by country.

This paper aims to build on these analyses by conducting an APC analysis of a 25-year time–series of cross-sectional surveys on alcohol consumption and abstinence in Great Britain. In particular, we seek to identify (i) whether an APC analysis indicates attributions of the UK population trend to rising female and youth consumption are justified and (ii) the demographic origins for recent increases in abstention. To facilitate attribution of effects to male and female trends, we separate our data to conduct gender-specific analyses.

## Method

### Data

The General Lifestyle Survey (GLF; formerly GHS or General Household Survey) is an annual cross-sectional household survey of approximately 20 000 individuals living in around 13 000 private households in Great Britain conducted by the Office for National Statistics. A probability-stratified two-stage sample design is used to draw a representative sample of private households from the postal address file. Household members aged 18+ were asked to complete the drinking section during face-to-face interviews from the GLF 1978 onwards, and from 1988 onwards 16- and 17-year-olds responded using a self-completion form. We obtained the GLF 1978 to 2009 data sets from the UK Data Archive (Supporting information, Appendix S1).

The questions to establish whether respondents abstain from alcohol have remained consistent; these are: (i) whether the respondent ever drinks alcohol nowadays and (ii) if not, whether the respondent means that they never drink alcohol nowadays or they drink alcohol only very occasionally.

Kemm 15 has summarized the questions for establishing drinking volume in the GLF 1978–98. Essentially, beverage-specific quantity–frequency (QF) questions were asked for respondents who drank very occasionally or more in the last 12 months. Over the years there have been changes to beverage categories (e.g. strong beer and alcopops were added in 1998) and QF categories (e.g. large and small cans of beer were introduced in 1990). From 1998 there were further changes to the QF questions (e.g. different wine-glass sizes were introduced in 2006). In 2006, the GLF updated their assumptions about beverage alcohol content used to convert quantity measures into alcohol units (1 unit = 10 ml ethanol) 25.

### Dependent and independent variables

The dependent variable in the abstention analysis is binary (1 = did not drink alcohol at all during the 12 months before interview). The dependent variable in the consumption model is the drinker's average weekly consumption in units.

The same set of independent variables was used for both the abstention and consumption analyses (Table 1). These are all categorical variables, namely age (16–17, 18–24, 25–34, 35–44, 45–54, 55–64, 65–74 and 75+ years), period (1980–84, 1985–89, 1990–94, 1995–99, 2000–04, 2005–09), birth cohort (19 5-year cohorts: from 1900–1904 to 1990–1994), household income (three groups: in poverty defined as below 60% of the annual median equivalized household income, high income defined as the top equivalized household income decile and medium income), education (four groups), ethnicity (six groups) and country (England/Scotland/Wales). Non-APC variables are included as controls for the APC effects, but the estimated coefficients are of interest in themselves. The APC effects and the effects of other independent variables are estimated simultaneously and control for each other. All models are fitted separately for men and women to obtain gender-specific estimates. Sensitivity analyses (SA) were performed which apply alternative groupings to APC variables and include gender dummies and interaction terms between gender and APC groups to test the robustness of the base case results.

**Table 1:** Estimated effects of demographic variables for men and women on alcohol abstention and drinkers' weekly consumption

		*Men*		*Women*	
		*Abstention*	*Mean volume*	*Abstention*	*Mean volume*
Household income					
	In poverty	**1.68 (0.05)**	**1.01 (0.02)**	**1.44 (0.03)**	**0.83 (0.01)**
	60% median to 90th percentile	Reference	Reference	Reference	Reference
	Top 10%	**0.60 (0.03)**	**1.11 (0.01)**	**0.75 (0.04)**	**1.22 (0.02)**
Education					
	No qualification	**1.81 (0.09)**	1.02 (0.02)	**2.18 (0.09)**	**0.87 (0.02)**
	Below A level	**1.18 (0.06)**	1.00 (0.02)	**1.22 (0.05)**	**0.94 (0.02)**
	A level	Reference	Reference	Reference	Reference
	Above A level	0.98 (0.05)	**0.92 (0.01)**	0.93 (0.04)	0.99 (0.02)
Ethnicity					
	White	Reference	Reference	Reference	Reference
	Asian—Pakistani or Bangladeshi	**100.66 (9.23)**	**0.46 (0.07)**	**121.74 (12.60)**	0.61 (0.18)
	Asian—Indian and others	**10.88 (0.67)**	**0.53 (0.03)**	**18.24 (1.01)**	**0.38 (0.03)**
	Black—Caribbean	**2.24 (0.26)**	**0.64 (0.04)**	**4.03 (0.31)**	**0.50 (0.03)**
	Black—African and others	**12.22 (1.17)**	**0.48 (0.05)**	**10.48 (0.85)**	**0.36 (0.05)**
	Others	**6.75 (0.52)**	**0.60 (0.03)**	**6.22 (0.38)**	**0.60 (0.04)**
Region					
	England	Reference	Reference	Reference	Reference
	Wales	**1.31 (0.09)**	1.04 (0.03)	**1.35 (0.07)**	0.96 (0.03)
	Scotland	**1.58 (0.06)**	**0.94 (0.01)**	**1.40 (0.04)**	**0.85 (0.02)**

Standard errors are reported in parentheses and statistically significant odds ratios (OR) and incident rate ratios (IRR) at the 5% significance level are indicated in bold type.

### Statistical methods

A logistic regression model is used to model the odds ratio (OR) for abstention. We excluded the GLF 1978, 1980 and 1982 from the abstention (and the consumption) analyses because ethnicity was not recorded. The sample size for the abstention analysis was 291 683, covering the period 1984 to 2009.

As in previous studies 20,23, we model the incident rate ratio (IRR) of drinkers' average weekly alcohol consumption using a negative binomial regression model. The final sample size for the consumption model was 200 144, covering the period 1986–2009 (excluding 1984, 2003/04, 2004/5 and 2007, as QF questions were not asked in these years).

In both analyses the reference groups were 45–54-year-olds, the 2005–09 period and the 1960–64 birth cohort, as these represent the most recent period and an average age or cohort group. Reference categories for other variables are shown in Table 1.

### Other model assumptions

The logistic and negative binomial models are fitted in Stata/SE version 10.1 software (StataCorp, College Station, TX, USA). The survey design was accounted for by using the commands for handling survey data in Stata. Survey weights were used for both the descriptive statistics and model fitting.

## Results

### Descriptive statistics

**Figure 2:**
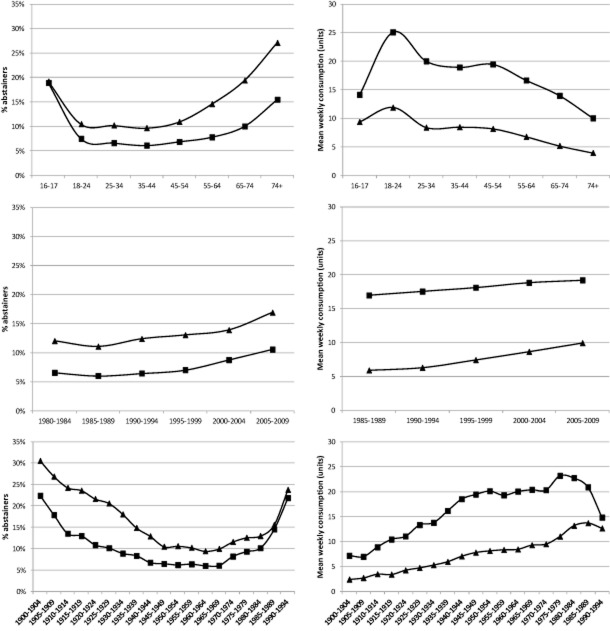
Mean proportion of abstainers and weekly consumption for drinkers by gender, age group, period and birth cohort. Squares: men; triangles: women

The weighted means of the proportions of abstainers and average weekly consumption for drinkers by gender and APC are presented in Fig. 2. By age, abstention rates are lowest at ages 18–54 (10–11% for women, 6–8% for men) and increase continually from age 55 onwards. By period, abstention rates increased by around 5% among both genders during the study period. By cohort, abstention rates fall from 22% in the 1900–04 cohort to 6–7% in the 1940–69 cohorts followed by a sharp increase in the proportion of abstainers among men born after 1985. Similar trends are also seen for women.

Drinkers' average weekly units peaks at age 18–24 (25.1 for men, 11.9 for women), falls by approximately 5 units and remains stable between ages 25–54, then declines into later life. By period, there are upward trends in drinkers' average consumption for both genders over the study period and the increase is greater for females. By cohort, we identify increasing consumption trends for male (from 7.1 units to 23.2 units) and female (from 2.4 units to 13.7 units) cohorts of drinkers up to those born before 1979 and 1989, respectively. Average consumption has declined in later birth cohorts, especially the most recent (1990–94) male cohort.

### Modelling alcohol abstention

**Figure 3:**
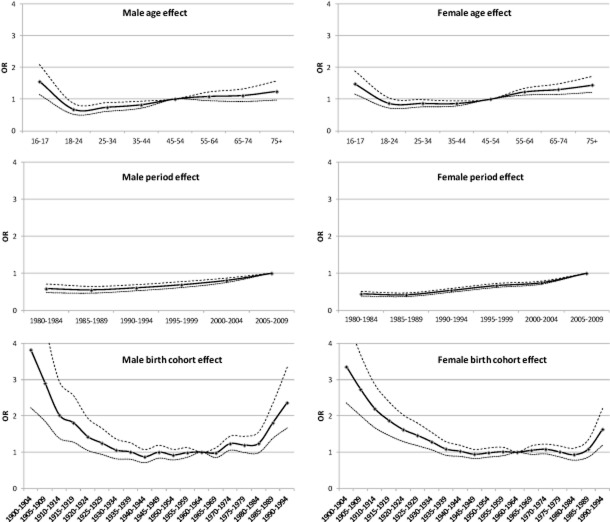
Age, period and birth cohort effects as odds ratio (OR) for men and women from logistic models predicting alcohol abstention. Reference groups are the 45–50-year age group, the 2005–09 period and the 1960–64 birth cohort. Dotted lines represent estimated 95% confidence intervals

The results from the logistic models are shown in Fig. 3 and Table A1 in the Supporting information, Appendix S2.

#### Age effects

Compared with the reference age group (45–54-year-olds), men aged 16–17 (OR = 1.55) and women aged 16–17 (OR = 1.48) or 55+ (OR = 1.23–1.44) are significantly more likely to be abstainers. Men aged 18–44 (OR = 0.67–0.82) and women aged 25–44 (OR = 0.86–0.86) are significantly less likely to be abstainers. The odds of alcohol abstention for men and women both decrease as people move from the 16–17- to the 18–24-year age group and the odds increase over their subsequent life-times.

#### Period effects

The odds of abstention have increased steadily from the 1985–89 period (OR = 0.55 male, 0.42 female) to the reference period of 2005–09.

#### Cohort effects

U-shaped curves were identified for cohort effects on abstention. Both men and women in the early birth cohorts (born before 1930) have significantly higher odds of abstention compared with the reference 1960–65 cohort. These ORs have wider confidence intervals due to the smaller sample sizes. For those born post-1930, odds of abstention compared with the reference cohort are significantly higher for men born between 1970 and 1974 (OR = 1.19) and, more markedly, between 1985 and 1994 (OR = 1.81–2.36) and for women born between 1990 and 1994 (OR = 1.63).

### Modelling drinkers' average weekly consumption

#### Age effects

**Figure 4:**
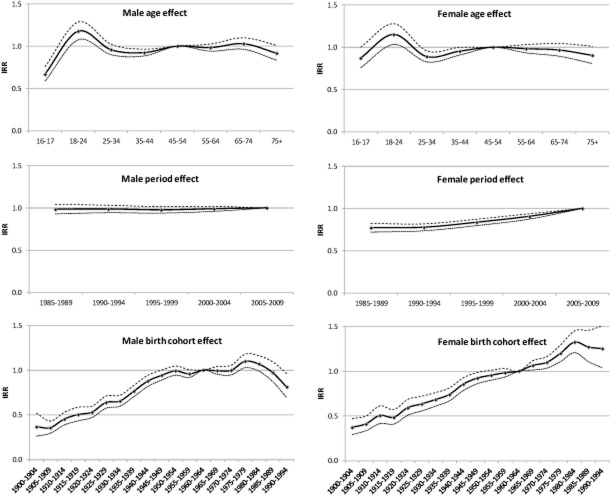
Age, period and birth cohort effects as incident rate ratio (IRR) for men and women from negative binomial models predicting drinkers' average weekly alcohol consumption. Reference groups are the 45–50-year age group, the 2005–09 and the 1960–64 birth cohort. Dotted lines represent estimated 95% confidence intervals

The results from the negative binomial models are shown in Fig. 4 and Table A2 in the Supporting information, Appendix S2. Consumption peaks for both men and women between ages 18–24 then drops sharply, but there is a rebound, particularly for women, of slightly increased consumption between ages 45–54. Compared with the 45–54 age group, both male and female drinkers aged 18–24 drink significantly more (IRR = 1.18–1.15), whereas men aged 16–17 and 35–44 (IRR = 0.67–0.92) and women aged 25–44 (IRR = 0.89–0.95) drink significantly less. No significant decline in consumption among drinkers was seen in those aged 55+ compared with the reference group.

#### Period effects

Male and female drinkers show very different period effects. No significant changes in consumption are identified for men, but successive significant increases are identified for women from 1990 to 1994 onwards (IRR = 0.77).

#### Cohort effects

Both male and female drinkers in the earliest birth cohorts (pre-1930) drink significantly less than the reference 1960–64 cohort. For those born post-1929, male drinkers born 1930–49 (IRR = 0.65–0.96) and 1990–94 (IRR = 0.91) drink significantly less than the reference cohort, while male drinkers born 1975–79 drink significantly more (IRR = 1.10). Female drinkers born 1930–49 drink significantly less (IRR = 0.68–0.92) and those born 1965–94 drink significantly more (IRR = 1.06–1.32) than the reference group, with the consumption level reaching its peak in the 1980–84 cohort. Following the generally upward trend in successive birth cohorts, there is a marked decrease of consumption level for male birth cohorts born post-1980, but the decrease is not significant for later female cohorts.

### Effects of other demographic variables

Table 1 presents the estimated effects and associated standard errors for other independent variables. Of note here is the finding that Scottish men and women are more likely to be abstainers and consume less than their English counterparts across the study period. We discuss this further in the study limitations.

### Sensitivity analyses

An alternative definition of abstinence was tested in an SA where people who drink very occasionally are also classified as abstainers; however, the results (not shown) did not differ substantively from the base case model. A range of one-way SAs applying alternative groupings to APC variables was performed (e.g. combining the 16–17- and 18–24-year age groups to increase sample size and number of surveys covering the youngest cohorts). Again, the results (not shown) did not differ substantively from the base case and the increasing probability of being an abstainer and decreasing consumption level for drinkers among later birth cohorts were still identifiable. Finally, gender dummies and interactions between gender and APC groups were tested and did not yield substantively different results.

## Discussion

The above results present a complex picture of the components of recent trends in UK alcohol consumption. Below we attempt to summarize the results by characterizing their contribution to the three broad components of the per capita consumption trend between 1986/87 and 2010/11 (Fig. 1).

### Relative stability (1986/87–1994/95)

Abstinence levels were generally increasing across men and women of all ages. However, the effects of this trend on per capita consumption appear to be cancelled out by rising average consumption among more recent cohorts who are at high consumption periods in the life-course compared with preceding cohorts.

### Upward trend (1994/95–2004/05)

The earlier birth cohorts who had higher abstinence rates and lower average consumption among drinkers were at the end of the life-course and were being replaced by more recent cohorts with lower proportions of abstainers and higher-consuming drinkers (particularly among females). Consumption among female drinkers continued a general rise during this period, although this effect was mitigated by ongoing increases in abstinence for both men and women.

### Downward trend (2004/05–2010/11)

The process of replacing earlier low-consuming, high-abstinence cohorts at the end of their lives with high-consuming, low-abstinence cohorts was largely completed, and smaller differences in abstinence rates were seen between the 1940s–80s cohorts who composed the bulk of the population. Drinkers in the most recent birth cohorts, who were also at the highest consuming point in the life-course, had lower average consumption levels than preceding cohorts, and these new cohorts also contained more abstainers. Abstinence rates also continued to rise across the population during this period, although female drinkers, again, also continued to drink more on average.

These findings suggest that recent UK per capita consumption trends are rooted in four phenomena. First, the United Kingdom appears to be moving progressively away from highly gendered drinking roles as changes in female drinking behaviour occur. Previous research has found similar shifts in some countries (e.g. United States, Finland) but not others (e.g. Germany, the Netherlands) 26,27, and findings from the GENACIS project suggest that this may be linked to changing attitudes to gender roles, increased female labour market participation, higher divorce rates and urbanization 28. Secondly, if behavioural change flows through social networks, as proposed by Skog 11, our evidence suggests that this appears to be primarily a generational process whereby successive birth cohorts adopt and adapt the behaviour of preceding cohorts, thus producing identifiable cohort trends. Although Skog's theory would predict temporally proximal cohorts to influence each other, as their social networks are likely to be well integrated, the influence should not simply be a transfer of behaviour to younger cohorts. If behaviour change was being transferred to older cohorts, this would dampen the cohort effects and produce period effects; there is little evidence of this in our results. A potential explanation for this may be younger age groups being more susceptible to behaviour change than older counterparts who have well-established drinking patterns. Thirdly, complex changes in rates of abstinence have occurred which can be summarized as general increases in abstinence across the population, but less so in pre-1940s cohorts and more so in post-1970s cohorts. The reasons for this are not well understood, but long-term immigration trends from high-abstinence cultures are an often-noted explanation when discussing our results with stakeholders. Further research is required to establish the causes of these trends, if they are occurring in comparable drinking cultures and what implications there may be for the cultural position of alcohol when such changes occur. Finally, a notable contributory factor to recent declines in per capita consumption appears to be declining average consumption and increased abstinence in the post-1985 birth cohorts; a finding seen in other UK surveys 29,30 and some, but not all, European countries 14. Again, the reasons for this are, as yet, relatively unexamined, although suggested explanations include increased economic pressures particular to this group, restrictions on youth-orientated retail and marketing practices, a cultural reaction against previous excesses, shifts to online recreation and increased focus on healthy life-styles 5,6.

Recent UK policy debate has focused on youth and female consumption. There is some justification for this, as our results show a clear spike in consumption in early adulthood and large increases in consumption among female drinkers. However, while early adulthood is associated with concerns regarding alcohol-related violence and disorder 4, a substantial proportion of the burden of alcohol-related harm also emerges from increased risks of chronic disease in later life 31. As the highest-consuming cohorts of female drinkers have moved past early adulthood and consumption among the most recent cohorts appears to be falling relative to their predecessors (albeit from a high baseline), greater policy attention should be given to the wider life-course and to population-level interventions. As many of the higher-consuming cohorts are yet to reach higher-risk ages for chronic diseases it should be recognized that, in the absence of interventions, rates of alcohol-related harm may not fall substantially for many years. While drinkers' consumption in these cohorts is likely to fall in line with our observed age effects, our results suggest it will remain at higher-risk levels than the cohorts that preceded them. More positively, our results suggest that rates of acute harms attributable to the heavy episodic drinking associated with youth consumption may show falls in recent years unless, as argued by some 6,32, a polarization is occurring whereby high-risk consumers are drinking more and low-risk consumers are drinking less. As we do not examine the distribution of consumption within demographic groups, we cannot speculate on this. However, a key priority for future work will be to assess the extent to which APC analyses permit accurate forecasting of consumption trends and resultant trends in harms.

### Limitations

There are a number of limitations to this study. Drinking patterns are not covered by the data, and our analyses do not permit examination of changes in the distribution of consumption within population subgroups. Therefore, we present only a partial picture of the components of the population-level trends. The estimated effects for both late and early birth cohorts are based on a small number of surveys and cover relatively few age groups; therefore, these estimates are less reliable than those for other cohorts and the interactions between APC effects are more difficult to quantify. In common with other surveys, the GLF underestimates per capita consumption relative to sales data 33. The impact of this on our analyses may be limited if underestimation is stable over time and across model covariates. However, we suspect that under-representation of heavy drinkers in Scotland explains our finding that Scottish consumption levels are lower and abstention rates higher despite robust evidence to the contrary. The response rate of the GLF has been declining over time, from approximately 81% in 1984 to 71% in 2009. The declining response rates may affect demographic groups differently to the detriment of the representativeness of the survey. Further research is required to quantify the impacts of declining response rates. Model identification presents a challenge for APC analyses, as birth cohort is a linear function of age and time period (i.e. cohort = period − age). Thus, exact linear dependence of the three variables occurs when aggregate tabulated data are used 34. This study follows others using similar data and groups the age, period and cohort properties of survey respondents into time intervals of different lengths 35. Different APC groupings were tested, as reported in the sensitivity analyses above, and the results of the base case APC models are also in line with the descriptive statistics shown in Fig. 2.

## Conclusion

The above APC analysis suggests that rising female consumption and the progression of higher-consuming birth cohorts through the life-course are key drivers of increases in per capita alcohol consumption in the United Kingdom. In contrast, recent falls in consumption appear attributable to reduced consumption and increased abstinence rates among the most recent birth cohorts, especially recent male cohorts, and general increased rates of abstention across the study period. These results support the use of policy interventions with the potential to impact upon adults of all ages, not just younger drinkers, to reduce substantially the rising rates of alcohol-related disease.

### Declaration of interests

None.

## Disclaimer

The General Lifestyle Survey data are Crown Copyright. Neither the Office for National Statistics, Social Survey Division, nor the Data Archive, University of Essex bears any responsibility for the analysis or interpretation of the data described in this paper.

## Supporting Information

**Appendix S1.** Data sources **Appendix S2.** Detailed model results
