# Supplementary material for: Trend analysis and modelling of gender-specific age, period and birth cohort effects on alcohol abstention and consumption level for drinkers in Great Britain using the General Lifestyle Survey 1984–2009
Source: Addiction. 2013 Sep 13;109(2):206–15. doi: 10.1111/add.12330 (PMC4016750; doi:10.1111/add.12330)
Supplement: Supplementary file 1 — Appendix S1. Data sources Appendix S2. Detailed model results [file add0109-0206-sd1.doc]

**Appendix 1: Data sources**

University of Essex. Institute for Social and Economic Research and National Centre for Social Research, General Household Survey, 1984 (GHS) [computer file]. Colchester, Essex: UK Data Archive [distributor], 2 December 1986, SN 2154.

GHS, 1986 [Computer file]. UK Data Archive [distributor]. 20 February 1989, SN 2569.

GHS, 1988–1989 [computer file]. UK Data Archive [distributor]. 20 November 1990, SN 2724.

GHS, 1990–1991 [computer file]. UK Data Archive [distributor]. 6 October 1992, SN 2937.

GHS, 1992–1993 [computer file]. UK Data Archive [distributor]. 19 January 1994, SN 3166.

GHS, 1994–1995 [computer file]. UK Data Archive [distributor]. 13 May 1996, SN 3538.

GHS, 1996–1997 [computer file], UK Data Archive [distributor]. 25 July 2002, SN 3804.

GHS, 1998–1999: Special Licence Access [computer file]. UK Data Archive [distributor]. 14 September 2009, SN 6271.

GHS, 2000-2001: Special Licence Access [computer file]. UK Data Archive [distributor]. 7 September 2009, SN 6270.

GHS, 2001-2002: Special Licence Access [computer file]. UK Data Archive [distributor]. 7 September 2009, SN 6269.

GHS, 2002-2003: Special Licence Access [computer file]. UK Data Archive [distributor]. 7 September 2009, SN 6268.

GHS, 2003-2004: Special Licence Access [computer file]. UK Data Archive [distributor]. 7 September 2009, SN 6267.

GHS, 2004-2005: Special Licence Access [computer file]. UK Data Archive [distributor]. 7 September 2009, SN 6266.

GHS, 2005: Special Licence Access [computer file]. UK Data Archive [distributor]. 14 December 2009, SN 6265.

GHS, 2006: Special Licence Access [computer file]. UK Data Archive [distributor]. 17 February 2010, SN 6264.

GHS, 2007: Special Licence Access [computer file]. UK Data Archive [distributor]. 17 February 2010, SN 6263.

General Lifestyle Survey (GLF), 2008: Special Licence Access [computer file]. UK Data Archive [distributor]. 12 May 2010, SN 6414.

GLF, 2009: Special Licence Access [computer file]. UK Data Archive [distributor]. 4 April 2011, SN 6737.

**Appendix 2: Detailed model results**

Table A1: Mean, standard error and p-value of the model coefficients of the logistic models for the age, period and birth cohort effects on alcohol abstention.

Note: OR - odds ratio.

Table A2: Mean, standard error and p-value of the model coefficients of the negative binomial models for the age, period and birth cohort effects for men and women on drinkers’ average weekly alcohol consumption.

Note: IRR - incident rate ratio.
